# A Case of Cancer of the Breast; with Remarks

**Published:** 1789

**Authors:** T. Hughes

**Affiliations:** Surgeon at Stroud-Water, in Gloucestershire


II. *A Case of Cancer of the Breast; with Remarks. Communicated in a Letter to Dr.* Simmons *by Mr.* T. Hughes, *Surgeon at Stroud-Water, in Gloucestershire.*

A Married woman, fifty-four years of age, in the month of July, 1783, desired my advice for a lump in her left breast. About a year before, she had, in falling, received a blow on this breast, which had been attended at the time with great pain: but the pain, as she informed me, soon afterwards went off, and she experienced no other consequence from the accident for eight months, when she perceived an induration, about the size of a hazel nut, which gradually increased in size, and became painful.

The tumour, at the time she applied to me, was rather above the nipple, of about the size of a walnut, and moveable; its surface rather flat, and its center very hard. The whole breast, which was small, was diminished in size. The uneasiness she felt was sometimes a dull, heavy pain in the part; but principally a great smarting, which had induced her once or twice to suppose the cuticle abraded. This smarting was not so much on the principal lump as a little lower down and more externally, towards the axilla, where, where, upon careful examination, was alſo found a ſmall moveable hardneſs: no ſoreneſs or ſmarting was felt when the ſkin was touched with the finger. She had been a ſufferer from cares and anxiety; and ſubject, but not in a great degree, to pains (which ſhe called rheumatic) in the limbs from her childhood. She had, likewiſe, ſince the diſappearance of the menſes, about five years before, been troubled at times with giddineſs, which had, however, lately left her, ſo that ſhe had for more than a year omitted her uſual blood-lettings. Her general health and complexion appeared to be good.

My opinion was, that the ſooner the tumour was removed the better; but as her family affairs did not permit the performance of an operation immediately, after taking away ſome blood and adminiſtering a purgative, I ordered in the mean time the cicuta internally, and externally an application ſimilar to that of Du Mare's tincture, recommended by the late Mr. Juſtamond.

Six weeks afterwards ſhe applied to me to have the operation performed. The tumour was but little enlarged, and was quite free from adheſion to the pectoral muſcles, but its center adhered to the ſkin, and I was concerned to find a

 cluſter cluster of indurated glands in the axilla, some of which lay high up. The pain in the breast was nearly as before, worse at some times than others, and it seemed, as she expressed it, " as " if there was something alive in it :" when the breast was most painful, the glands in the axilla were sore and uneasy. She had noticed the glands in the axilla about three weeks ; and before that time she had often observed a numbness in the arm.

I requested Dr. Snowden to see her. The result of a careful examination was, that, although we were apprehensive of a return of the disorder, it would be better to take the chance of the operation than leave the disease to take its course; as in all probability ulceration would, in that case, soon take place, with all its consequences; and this being represented to her, she determined to abide by the resolution she had before taken.

A spare diet having been directed, and a gentle purge given, I performed the operation on the 15th of September, in the presence of Dr. Snowden, who afterwards visited her occasionally, and assisted with his advice. For reasons which shall be given by and by, I had determined to conduct it in such a manner as to attempt an union union of the wound by the firſt intention. Although the tumour of the breaſt was ſmall, on account of the indurated glands in the axilla and the ſmall lump betwixt them and the principal tumour, I thought it beſt to make but one wound, ſufficiently extenſive to admit of the removal of every diſeaſed part without embarraſſment; and the adheſion of the tumour to the ſkin was ſo very cloſe to the nipple, that it was not prudent to ſave it. Therefore, beginning the firſt inciſion at the axilla, it was carried on with a ſweep below the nipple near to the ſternum, from whence the ſecond inciſion was begun, carried above the nipple, and terminated in the firſt on the ſide of the breaſt towards the axilla; the ſkin in the center betwixt the two inciſions being about an inch wide, and the angles, where the inciſions met, being made very acute, to prevent future puckering of the ſkin. The principal tumour and the ſmall lump on the ſide of the breaſt were then readily diſſected out, together with the remainder of the glandular and fatty part of the breaſt; and afterwards the glands, to the number of four or five, were cautiouſly removed from the axilla, with a conſiderable quantity of fat and cellular ſubſtance. Upon careful examination, every diſeaſed part

 appeared appeared to be removed. During the operation the blood ſprang from two or three arteries, of which the moſt conſiderable was one in the axilla, but was ſuppreſſed by the preſſure of a finger ſo as not to flow even after waiting ſome time after the parts were removed. The lips of the wound were eaſily brought together; and, to retain them in contact, five ſtitches of the interrupted ſuture were employed, viz. one at the junction of the two inciſions at the outſide of the breaſt, three betwixt that and the ſternum, and one betwixt the junction and the top of the inciſion in the axilla. Betwixt the ligatures the lips of the wound were covered with lint preſſed out of balſ. traumaticum; and the axilla and breaſt with graduated compreſſes, wet with a ſaturnine ſolution, and ſuſtained by a bandage fixed with tapes on the oppoſite ſide: that part of the bandage covering the affected ſide was compoſed of linen, through which the ſaturnine ſolution was afterwards frequently ſoaked; the remainder was of flannel.

For three or four days after the operation ſhe had a quick pulſe, but ſuffered chiefly from lowneſs, having little or no ſymptomatic fever, except for a few hours in the afternoon of the 18th, after a little irregularity in diet. An opiate opiate was given every evening till the 19th, not to eafe pain, but to calm an agitation of fpirits; and, after the firft two days, a moderate allowance of wine, a faline cordial mixture, and a cordial joined with the opiate, fuited her better than the low diet fhe was put upon at the time of the operation. Except in the afternoon of the 18th, as juft mentioned, fhe fuffered very little pain, and that chiefly in the axilla, when the arm was accidentally moved or not properly fupported, or when the compreffes were not kept cool by frequently applying the faturnine folution.

On the 17th there was a very flight rednefs and fwelling, without tenfion, juft above the middle of the wound at the breaft.

On the 19th, obferving a little difcharge, the lint, after being firft foftened with vinous fpirit, was removed; the lips of the wound were found very little diftant from each other in any part; in moft places in contact; anteriorly correfponding to that part where the principal tumour had been removed: and where the rednefs and fwelling were obferved, two or three tea-fpoonfuls of thin pus were difcharged through a chafm of half an inch in length, and the matter being confined, one of the ligatures was removed and another ſlackened; the pus in other parts was thick, and barely ſufficient to moiſten the lint. The wound was dreſſed with lint moiſtened with balſ. copaivi, and the compreſſes with the saturnine ſolution were continued. She ſat up an hour this day.

On the 20th, except the part juſt mentioned, and a ſmall opening at the axilla, by which a little pus was preſſed out, the wound was united. All the ligatures were removed, by none of which was the ſkin cut; a ſufficient proof of there having been no tenſion. The ſaturnine application was not required to be ſo frequently renewed. The Peruvian bark was now given, and continued for ten days.

On the 21ſt ſhe ſat up ſeveral hours, and even walked down ſtairs.

On the 23d, the matter lodging under the lower lip of the wound anteriorly, a counter-opening was made about an inch below the wound with the point of an abſceſs lancet, and a ſuitable cotton ſeton introduced: the diſcharge was ſo much leſſened by the 28th as to render its longer continuance unneceſſary.

On the 24th the ſaturnine ſolution was diſcontinued. A large fontanel was opened in the thigh.

On

On the 25th, from the axilla, where remained of the wound about an inch and a half in length and one tenth of an inch in breadth disunited or unhealed, was a considerable discharge of lymph, which was suppressed by the usual applications in similar cases; and in a few days the wound was quite cicatrized. Before this took place some pimples appeared on the neighbouring parts, which occasioned a slight discharge, excoriation, and itching, and which continued for some time, so as to require some attention and dressing; but this was so trivial a circumstance, that it would have been unnecessary to have mentioned it, were it not for the propriety of stating the case accurately. Considered as a mark of acrimony, small doses of calomel were given for some nights, interposing now and then a dose of Glauber's salt.

By the latter end of October she was so free from pimples as not to require any application; she had recovered her general strength; daily gained strength and freedom in the motion of the arm, which was at first considerably confined by a tightness at the anterior armplait, and was free from a soreness she had felt on the inside of the biceps muscle before the operation was performed. The mark left by the wound was a mere mere line, with a fmall depreffion where the principal tumour had lain.

She was advifed to be attentive to maintain a difcharge from the iffue; to confine herfelf to as mild a diet as poffible; and once or twice a year to take the calomel for a week or two as before. Thefe rules, with an occafional blood-letting or two, were pretty well, though not ftrictly, obferved.

She never recovered the ftrength of the arm fo perfectly as before the operation, yet fufficiently to be often employed in occupations requiring confiderable exertion. In the fpring of the next year fhe was alarmed with pains about the original feat of the tumour, fhoulder, and arm, with a ftiffnefs and forenefs in the axilla, and with languors, to which fhe had been before fubject in hot weather: the pains feemed to her to be not in the foft parts, but in the bones. Thefe fymptoms went off in about a month. She had flighter returns of them for a few days, at times, about the autumn of the year 1785. She thought that they were brought on or increafed by taking cold, by heat, or by much exertion in her employments. Sometimes fhe felt the pains fhooting down to and going off by an increafed difcharge from the iffue, and obſerved that now and then the diſcharge was very fœtid for two or three days, without any neglect in dreſſing it.

Except theſe complaints, ſhe enjoyed good general health, and there appeared no evident marks of a return of the diſeaſe till the latter end of the year 1786, when, beſides the above ſymptoms, ſhe began to ſuffer pains in other parts of the body, and two ſmall indurations were diſcovered in the courſe of the cicatrix in the axilla, one of which was cloſe to the arm, and a third more forwards and a little above the cicatrix: upon this laſt the ſkin moved freely; but this was not the caſe with thoſe in the axilla. The indurations in the axilla ſlowly increaſed, and her general pains, like rheumatiſm, alſo, ſo that before the cloſe of the year there was no part of her body, except the head, but at times ſuffered; the *right* arm was more affected than the *left*, and at the indurated parts ſhe had now the ſame ſenſation of ſomething alive, as formerly, at the breaſt. At this time the iſſue was in a great meaſure healed.

A ſeton was made in the ſide, about a hand's breadth below the cicatrix. For ſome weeks ſhe took flores martiales, or cicuta; a remedy ſimilar to Du Mare's tincture was applied to

 the the indurations ; and an hemlock bath was uſed twice a week. She did not appear to receive any benefit from theſe, except the bath, by which her general pains ſeemed to be abated; but her ſtrength gradually decayed, ſhe had frequent languors, now and then pain through the cheſt affecting reſpiration, once or twice colic pains, and at laſt difficulty in ſwallowing ſolid food, and pain of her ſtomach upon taking her pills or any thing ſolid ; and ſome time after the diſuſe of the bath the pains of the limbs increaſed again ſo as to break her reſt. During this ſtate a large dark-coloured puſtule broke out in the forehead, and continued in nearly the ſame ſtate for ſome weeks before it died away ; the diſcharge from the ſeton too turned the ſilk black, raiſed pimples, and excoriated the ſkin to a conſiderable diſtance.

Although the indurations in the axilla increaſed, they never ulcerated, nor acquired any conſiderable ſize ; they occaſioned little or no pain, and ſhe once obſerved that the ſenſation of ſomething alive was ſhifted from them to the ſeton : the ſmaller anterior induration did not increaſe, and the remainder of the cicatrix continued ſoft, ſmooth, and moveable. After this ſhe took no other medicine than a laxative one, to obviate coſtiveneſs, which required it frequently.

On the 23d of April, 1787, when, from her feeble ſtate, ſhe appeared not able to ſurvive many weeks, ſhe fell backwards from the top of the ſtairs to the bottom, and received a contuſed wound on the poſterior part of the left parietal bone, which bled freely, but not profuſely, and another contuſion on the anterior part of the right parietal, beſides other ſlighter bruiſes.

A few hours after this accident her ſpeech left her, ſhe gradually ſunk, and died about thirty hours from the time of the fall. Mr. Williams, Surgeon at Durſley, who viſited her after the accident, informed me, that he obſerved no ſymptoms that could be attributed to miſchief done to the head, and he was of opinion that the fall proved fatal to her only from her infirm ſtate.

While living ſhe had expreſſed a deſire that I might open her after her death, which I accordingly did two days afterwards, in the preſence of Mr. Williams, and Mr. Taylor, Surgeon at Wotton-Underedge. The appearances on diſſection were as follows: Juſt above the ſmall curvature of the ſtomach was an indurated gland, of about the ſize of a large French bean. There were one or two ſimilar but ſmaller ones in the meſentery; and a ſmall cluſter of ſtill ſmaller ones in the meſocolon, on the left ſide. In the cellular ſubſtance covering the anterior ſurface of the left kidney were ſome ſmall hard bodies, feeling like ſhot or roundiſh ſeeds under the finger. The abdominal viſcera were ſound. In the thorax the lungs were very ſound, (except a ſmall ſtony ſubſtance or two, like ſhot, found near their ſurface) and had contracted only a ſlight adheſion at the upper part of the left lobe. The heart was ſmall for the ſize of the body, and cut rather more tender than uſual, without any marks of putrefaction. On its under ſide was here and there a white tendinous-like appearance, running in lines, flat and narrow, and ſomewhat in the direction of the veſſels, but ſtraighter, probably the beginning of oſſification. On one ſide of the aorta, a little below the curvature, was a pretty broad oſſification: at the curvature were four trunks, the unuſual one ſmaller than thoſe of the left carotid and ſubclavian, betwixt which it ſprung. The induration at the axilla did not extend into the cheſt or towards the clavicle, no traces of it being found there. Under the ſcalp at both contuſions was a conſiderable considerable ecchymosis; but no fracture, nor any extravasation or appearance within the scull which could be considered as morbid, there being only a very small quantity of water in the ventricles and on the anterior part of the beginning of the medulla spinalis.

REMARKS.

That the tumour in the breast of our patient was cancerous cannot be doubted; that by the removal of it she enjoyed life three years longer than probably she would otherwise have done may perhaps be allowed; and it may be granted likewise that the symptoms of a cancerous diathesis, independent of the small indurations in the axilla, wore out her strength and proved fatal; for the fall cannot be considered as accessory to her death, any otherwise than as having somewhat shortened the duration of a frame too weak to bear its shock.

The number of dissections of cancerous cases hitherto given to the world is comparatively small; and in all that I have read, where the progress of the disease, in an external part, did not seem sufficient to occasion death, some viscus, gland, or other part, situated internally, was found affected with the disease. I confess my expectation was was to have met with ſome ſuch appearance in this patient; but her death cannot be aſcribed to any ſuch cauſe. The ſmall glandular ſwellings in the abdomen were probably not at all painful in themſelves, and certainly could not occaſion any injurious preſſure on other parts. This, therefore, may be conſidered as a proof of the opinion, "that cancerous acrimony may pervade "certain ſeries of veſſels, and create pains, "without ſeizing upon any part with ſuch violence as to deſtroy its functions *."

The ſucceſs attending ſome late attempts to procure an union of the wound by the firſt intention after amputation of the limbs †, and my having ſucceſsfully proceeded upon this principle after the removal of one or two encyſted tumours, induced me to extend the ſame practice to the above caſe; but what principally determined me was the conduct of Mr. Wilmer ‡, in the firſt operation performed on one of his patients for the removal of a ſchirrus of the breaſt, where he approximated the ſides of the

* Fothergill. Medical Obſervations and Inquiries, Vol. V. art. 14.

† Alanſon's Practical Obſervations upon Amputation.

‡ Caſes and Remarks in Surgery, Caſe 27.

wound with flips of fticking plafter. Mr. Wilmer, indeed, feems to have apprehended, that this method contributed to a return of the difeafe in that patient; but the event of the fecond operation, conducted in the oppofite method, did not prove his fears to have been well founded; and that they were groundlefs I was fatisfied, by the different event of thofe cafes of cancer of the lip which had, and of thofe which had not, been treated in the method of the harelip *. I thought I had improved Mr. Wilmer's method, not knowing at that time that the fame plan had been purfued by any other practitioner of the prefent day; but fome months afterwards I learnt that Mr. Fearon † had treated two cafes in a fimilar manner. In thefe, Mr. Fearon, as well as Mr. Wilmer, employed fticking plafter without future; and he fays ‡, that, in his later practice, he never ufes ligatures, finding the adhefive plafter fully fufficient: but I think with Mr. Bell §, that ligatures are preferable. It is true that the infertion of the ligatures gives

* Bell's Treatife on Ulcers, page 273.

† London Medical Journal, Vol. IV. page 406.

‡ Treatife on Cancers, Ed. 2, page 81.

§ Syftem of Surgery, Vol. II. page 456.

fome additional pain; but this would be very little if the ftraight needle was ufed, which might here be done very conveniently *, paffing it through each lip of the wound, from within outwards, as advifed by Mr. Bell †.

It deferves to be confidered, however, when we proceed upon this plan, whether or not the lips of the wound fhould be brought into contact in every part by future or otherwife. Mr. Fearon obferves ‡, that fuppurations have happened in confequence of inattention to exclude all the air under the teguments, by bringing them carefully into contact with the fubjacent parts. That the confinement of air will have this effect there can be no doubt; and I apprehend that this may fometimes depend upon another cir-

* From the difficulty or impoffibility of ufing a ftraight needle in fome fituations, the crooked one was undoubtedly contrived; and from the ufefulnefs of the latter in thefe furgeons feem to have adopted it in other fituations, where the ftraight one (which they have confined almoft to the fole purpofe of fewing up a dead body) might be more conveniently ufed, In July, 1787, a cafe of a very extenfive, irregular wound of the face occurred, in which I had occafion to apply feven futures; all of which, except one at the internal canthus of the eye, were made with the ftraight needle.

† Syftem of Surgery, Vol. I. chap. 1. fect. 2.

‡ Treatife on Cancers, page 81.

cumftance cumſtance, to be preſently mentioned. Although a more ſpeedy union could hardly take place, than in our patient, throughout the greateſt extent of the wound, yet a ſmall ſuppuration happened in about one tenth of it. Had one of the ſtitches been omitted, the neceſſity of making a puncture, though a ſmall inconvenience, might not have occurred; much, therefore, will depend on the judgment of the ſurgeon, at the time of operating, to determine to what extent it will be proper to proceed on the uniting plan. It is probable that it may more fully take place in caſes where the tumour is extenſive, and free from tuberous projections on its poſterior part, ſo as to make a pretty equal preſſure on the pectoral muſcle, than in thoſe where the tumour is ſmall, or large, and having any ſuch projection, by its preſſure may have produced partial waſting and impreſſion in the muſcle; in which caſe the lips of the wound, being brought into contact, will be, in a manner, ſuſpended over the muſcle. This circumſtance, of impreſſions in the pectoral muſcle, is little noticed by writers *; nor is it material, if the wound be treated in

* M. Faget obſerved it in a caſe where he amputated the breaſt:—" Elle avoit quelques éminences en forme de mamme-" lons in the old way, by filling it with lint, &c.; but if treated in the contrary method, it is a matter of importance. In ſuch parts it may be beſt not to bring the lips of the wound into contact, but to apply ſuitable compreſſes only, or make a ſmall longitudinal inciſion of the inferior portion of the teguments, as adviſed by Mr. Bell *. In ſhort, it muſt be remembered, that we are to procure, if poſſible, an union of the teguments with the ſubjacent parts, and alſo of the lips of the wound with each other; that caſes may be ſo favourably circumſtanced as that both intentions may be accompliſhed at the ſame time; but if otherwiſe, that the latter muſt give place to the former.

I have been the more particular in pointing out the ſmall ſuppuration in my patient, from an apprehenſion that ſimilar circumſtances in other caſes of the ſame kind may contribute to a diſuſe of this method of operating in favour of the old practice, it being well known that practitioners

"lons qui s'enfonçoient un peu dans le muſcle pectoral, & que "j'aurois peut-être coupées, ſi je n'avois pas eu ſoin de tirer "la tumeur vers moi & de la detacher preſque entierement "avec les doigts." Memoires de l'Academie de Chirurgie, Tom. I. p. 683.

* Syſtem of Surgery, Vol. II. page 444.

titioners are too apt to difcard modes of treatment which do not equal their moft fanguine expectations in every point; and I am the more inclined to this opinion, as I find that the fame plan has been ufed, in a greater or lefs degree, by fome of our forefathers, and which perhaps, from fome fuch caufe, might not have been generally adopted.

The approximating or uniting the fides of wounds, practifed by the ancients * and fome of the moderns, after the extirpation of encyfted and other tumours in various parts of the body, have been ufed by a few of the latter after that of the fchirrus of the breaft and teftis. After the amputation of the breaft, Wifeman † employed a crofs ftitch; Purmannus ‡ flips of fticking plafter; and Mr. Hill § ufed futures in one of his patients. Garengeot was a warm advocate for this method: he urged it by the fame arguments which have been lately ufed, drawn from the fuppofition of the difeafe being originally a local one, and from the greater pro-

* See Celfus de Medicina, Lib. 7, cap. 6.

† Chirurgical Treatifes, Book 1, chap. 21, obf. 9.

‡ Chirurgia Curiofa, Eng. Tranflation, Book 2, chap. 3.

§ Cafes in Surgery, page 16.

 bability bability of its again attacking the wound treated on the ſuppurative plan; in order to promote a ready union of the wound, he adviſed the inciſion of the teguments to be made in a ſuitable manner, and that ſutures, if the lips of the wound can be brought into contact, or, when that cannot be accompliſhed, ſticking plaſter ſhould be employed *. He ſpeaks of its being very ſucceſsfully practiſed by able ſurgeons; yet we do not find it mentioned by his contemporaries

* " Il ne faut pas s'embarraſſer ſi la peau eſt coupée exactement *en rond*; c'eſt même une faute que cette grande exactitude; car ſi on veut une plus prompte reünion, c'eſt de faire une plaïe *en long* afin d'avoir plus de facilité à approcher les bords de la peau, pour en faire la ſuture ſi la choſe eſt poſſible; ce que d'habiles chirurgiens ont fait avec un très grand ſuccès, même à des cancers tous ulcerés.

" Si l'on fait cette opération dans un endroit où la peau ne prete pas beaucoup, & que la perte de ſubſtance ſoit ſi conſiderable, qu'on ne puiſſe pas approcher les deux lévres de la plaie l'une contre l'autre, pour en faire la ſuture; il eſt d'un chirurgien qui tend à guérir promptement, de ſe ſervir de l'emplâtre d'*André de la Croix*; car ſi on ne peut pas faire tout le bien qu'on ſouhaite, qui eſt de reünir exactement la diviſion, il faut du moins en approcher le plus qu'il eſt poſſible.

" On raries and countrymen, Le Dran and Petit *. De la Faye †, indeed, in his remarks, orders the ſides of the wound to be brought together as nearly as poſſible, and compreſſes to be applied ſo as to retain them in that ſituation; but his inciſion, as well as that of Wiſeman and Purmannus, being circular, the lips of the

" On voit, par notre méthode de traiter toutes les tumeurs " enkiſtées §, que nous ſommes fort éloignés du ſentiment de " ceux, qui pour purifier la maſſe du ſang, & prévenir par " conſequent la récidive, entretiennent une longue ſuppura- " tion, pour ſervir, à ce qu'ils diſent, d'egout aux ſeroſités " mauvaiſes dont les chairs & les graiſſes ſont infiltrées: il " ſeroit même à ſouhaiter de pouvoir reünir la plaie en vingt- " quatre heures, car il y a lieu de croire que la récidive de ces " maladies ne vient qu'en conſéquence des longues ſuppura- " tions, ou une partie du pus eſt priſe par les vaiſſeaux ſan- " guins, & portée dans la maſſe, pendant que l'autre ſort à " tous les panſemens qui ſont trop frequens, & dans leſquels " on ſe pique trop d'exactitude." Traité des Operations de Chirurgie, 2d. Edit. Tom. II. p. 421.

* Heiſter (General Syſtem of Surgery, Engliſh Tranſlation, Part II. chap. 107.) mentions it as propoſed by Petit; but nothing is ſaid of it by the latter in his poſthumous work, Traité des Maladies Chirurgicales, although he treated encyſted tumours in this way.

† Cours d'Operations de Chirurgie par M. Dionis, 4th Edit. p. 465.

§ He reckons cancers among the encyſted tumours.

wound wound could not be brought into contact. Heifter * mentions his having followed Garengeot's method in one inftance, and that the wound was foon healed ; bnt the difeafe returning foon afterwards, he feems to have been difcouraged from ufing it again.

It appears to have been the general method with the ancients †, after performing any operation in the groin or fcrotum, to apply the fibula or future to the wound. Bonetus ‡ relates, from Bartholine, an inftance of farcocele removed by caftration ; after which the furgeon fewed up the wound : and this is not mentioned as an unufual practice. Purmannus § directs, after removing the carnous fubftance || in a farcocele, to few up the wound with two or three ftitches ; which he did likewife in a cafe of hydrocele, after having made an incifion. Sharpe ¶ fays, that it will greatly facilitate the cure of the wound to pafs one or more ligatures. Although

* General Syftem of Surgery, Part II. chap. 107.

† Celfus de Med. Lib. 7, cap. 19.

‡ Sepulchret. Anatom. Lib. 3, fect. 29, obf. 22, §. 2.

§ Book 2, chap. 14.

|| He confounds the fchirrous teftis with other difeafes of the part, as did other authors of that time.

¶ Treatife on Operations, Chap. 10. Critical Inquiry, Chap. 3.

Mr.

Mr. Sharpe's treatifes have been held as a ftandard by moft practitioners in England for near half a century, I believe that his directions in this refpect have been rarely followed. Mr. Fearon has the merit of bringing the uniting plan in this inftance, as well as in that of the fchirrous breaft, into more general ufe; and it is pleafing to fee it now adopted after other chirurgical operations.

There are fome other parts of the treatment which I think deferve notice. In my patient it is feen that lint moiftened with balf. traumaticum was applied over the edges of the wound. This application I have for a long time ufed to wounds that were to be united by the firft intention, as did our forefathers, and do the common people; but it feems not to be in general ufe with furgeons. It excludes air, impedes fuppuration, by drying and adhering to the fkin becomes affiftant to the ligatures, and is not loofened by any watery application coming into contact with it that may be thought proper to be ufed *. My patient found great relief from the frequent

* I think the method of ufing it is improved by infpiffating it to the confiftence of honey, and fpreading it pretty thick upon fine old linen or filk: it may be permitted to dry, cut frequent renewal of the ſaturnine ſolution, as did a patient whoſe caſe is related by Mr. Wilmer *, although the latter did not in that inſtance proceed upon the uniting plan. We both applied it cold, being in ſummer; but under ſome circumſtances, perhaps, it may be better to uſe it lukewarm. By thus keeping the parts cool, both hæmorrhage and inflammation will be more effectually prevented.

A bandage, made on the ſame principle as the napkin and ſcapulary, is much more commodious than the roller †. Flannel is certainly preferable to linen; but if the compreſſes are to be kept wet, it is beſt that that part of the bandage which covers them be of linen, that the liquid may be ſoaked through it without diſturbing the bandage.

cut into a proper form at the time of uſe, and rendered adheſive by moiſtening its ſurface with vinous ſpirit, as we do court plaſter with water. In this way, at leaſt, the ſmarting, which the application of it in its liquid ſtate is apt to excite, may be prevented.

* Caſe 26.

† Mr. Hill's bandage for the thorax (Caſes in Surgery, page 222) is a good contrivance. I have for near twenty years uſed a ſimilar one, with this difference, that it has loops on one ſide, and on the other double ſtrings of tape, by which means it is more eaſily tightened or ſlackened occaſionally.

The remarks here made on the chirurgical treatment are ſubmitted with deference to thoſe gentlemen whoſe ſituation obliges them to a frequent performance of theſe and ſimilar operations. If we ſhould pride ourſelves in thinking that we have been making great improvements, by deviating from the general practice of our more immediate predeceſſors, we may be humbled by the reflection, that we have done little more than laid aſide ſome bad practices imbibed from them, and returned to the plan made uſe of by the ancients; not in cancers, indeed, for they conſidered them to be ſo liable to return, that Celſus has not left us any directions for removing them, except the ſarcocele, which he does not reckon with the carcinoma; but in wounds in general, as well as in thoſe occaſioned by the removal of encyſted tumours, if circumſtanced ſo as to admit of union or approximation, they applied the ſuture or fibula, ſome agglutinative medicine, and a ſponge ſqueezed out of vinegar, wine, or cold water, which was carefully renewed, ſo as not to ſuffer the part to become dry *.

* Celſus de Med. Lib. 5, cap. 26. Lib. 7, cap. 6.

Permit me to add a few other remarks which have occurred on confidering this fubject. The bad fuccefs which the late Profeffor Monro* met with in the extirpation of cancers is well known, and appears to be very fatisfactorily accounted for †; but it is rather furprifing to fee Sharpe and Le Dran mentioned ‡ as dif-couraging furgeons from extirpating cancers without diftinction. Sharpe, fpeaking of the fchirrous and cancerous breaft, allows that the operation is precarious, from the great dif-pofition in the conftitution to form a new can-cer; but mentions fome circumftances under which he thought it had beft fucceeded, and in which he not only allowed but even encou-raged the operation §. Le Dran took great pains to afcertain the cafes in which fuccefs

* Medical Effays of Edinburgh

† Bell. Treatife on Ulcers, Part II. fect. 8, §. 2.

‡ Hill's Cafes, page 2. Fearon's Treatife, page 3.

§ " There are fome furgeons fo difheartened by the ill fuc-" cefs of this operation, that they decry it in every cafe, and " even recommend certain death to their patients rather than " a trial, upon the fuppofition it never relieves; but the in-" ftances where life and health have been preferved by it are " fufficiently numerous to warrant the recommendation of " it." Treatife on Operations, Chap. 26.

might be expected from extirpation; for that he indiscriminately condemned it cannot be inferred from what he has said in his Treatise of Operations; and whoever will peruse what he afterwards published in another work *, will find sufficient encouragement to operate.

On the other hand, from Mr. Hill † having not a seventh part, who were not cured, and who suffered relapses after the healing of the wound, out of eighty-eight patients on whom he operated, some gentlemen ‡ seem to infer, that success may be expected to attend the extirpation of cancers, wherever situated, without distinction; a conclusion which the premises do

* Memoire avec un precis de plusieurs observations sur le cancer. *Vide* Mémoires de l'Académie de Chirurgie, Tome III.—In the second section of that Essay, after relating some cases of cancer of the breast in which relapses happened after the operation, he says, " Mais faut-il pour " cela, refuser tout secours, & ne pas essayer de guérir par " une operation qui a si souvent réussi, lorsqu'il n'y a que " de la probabilité pour la récidive? Il faudroit avoir une " certitude physique de l'impossibilité de la réussite; & c'est " là le seul cas où on ne doit pas faire l'operation."

† Cases in Surgery.

‡ Bell. Treatise on Ulcers, Part II. sect. 8. — Duncan. Medical Cases, page 111.

by no means juſtify, and which, I believe, would hardly have been drawn by Mr. Hill himſelf: for if we inquire farther into the matter, we find that of the whole number there are only five inſtances of cancer of the breaſt, two of which were not cured, and another patient ſuffered or was threatened with a relapſe; ſo that the ſucceſsful caſes in the breaſt were only two in five: whereas the other eighty-three, with very few exceptions, were cancers ſeated chiefly in the ſkin, and eſpecially the lip, and in theſe the ſucceſs riſes in proportion as it is reduced in thoſe of the breaſt, being more than ſeven-eighths of the whole.

It would be a curious, if not a uſeful inquiry, to aſcertain the proportional ſucceſs attending the extirpation of cancers in different parts of the body; but it may be doubted whether the practice of any individual, however extenſive, is ſufficient for this purpoſe; therefore it would be beſt done by the joint obſervations of many. From the hiſtories of caſes already publiſhed, not given in a regular ſeries, an abſolute concluſion cannot be drawn, more eſpecially as writers have been too fond of relating their ſucceſsful caſes only, or of publiſhing them before a ſufficient period has elapſed after the operation:

ration; ration: but perhaps we ſhall make ſome approaches towards the truth by examining how they ſtand in this view, or what is the proportion of ſucceſsful caſes to thoſe which either entirely failed, or in which relapſes happened ſooner or later after the healing of the wound.

Of the caſes which I have met with, being upwards of two hundred, and taken as they occurred, without ſelection, I obſerve, that in cancers affecting the ſkin chiefly, (of which there are four in the lip or other parts of the face to one in all the other parts of the body) ſucceſs has been procured in about four-fifths. In the eye, the ſucceſsful caſes are more than one half: in the mouth, about two-thirds: in the breaſt, they hardly exceed one half: in the teſtis, about four-ſevenths: in the penis, they equal or exceed thoſe of the ſkin: in the limbs, if not confined to the teguments, not more than one-third.

Theſe differences are ſurely too ſtriking to be attributed to accident, and ſhould, therefore, influence the conduct of the ſurgeon. In cancers of the ſkin, the above-mentioned proportion does not riſe ſo high as in Mr. Hill's caſes, which may be attributed partly to the diſeaſe being formerly ſuffered to proceed to a greater length length before removal than latterly, and partly to the better method of treating the wound used of late years than formerly. M. le Dran's conclusions * on this head will appear just; and they are now farther confirmed by the success attending the extirpation of the cancer of the scrotum †, and of a species of cutaneous cancer prevalent in another part of the world ‡. Cancers of the penis seem to be as local as those of the face, or other external parts. But although we may allow that the cutaneous cancer is for the most part local, we cannot conclude the same with regard to that of the breast, testis, and other parts seated below the skin: that of the testis is probably oftener local than that of the breast; of which latter the small successful proportion from extirpation must be referred to the sex and time of life in which it usually makes its appearance.

Practitioners, for the most part, recommend early extirpation; which advice is undoubtedly good with regard to schirri and cancers in gene-

* Memoires de l'Academie de Chirurgie, Tome III.

† Pott. Chirurgical Observations relative to the Cataract, Polypus of the Nose, Cancer of the Scrotum, &c.

‡ Moseley. Treatise on Tropical Diseases.

ral: but M. le Dran *, from the event of some cases, concluded that the operation is improper, in those occasioned by the cessation of the menses, until many years are elapsed, and while the tumour increases without pain; judging that in such nature is making a critical deposit, and that if before it be compleated she is interrupted a fresh one will be made. Although it is now thirty years since this remark was given to the world, I do not know that any regard has been paid to it: but certainly, from the known accuracy and solid judgment of M. le Dran, and the facts adduced, it deserves attention; and it would be right, by future observations, to determine whether it be well founded or not. Was it of such patients that Mr. Sharpe † said, "when a schirrus has admitted of a long delay "before the operation, the patient seems to "have a better prospect of cure without danger of a relapse, than when it has increased "very fast and with acute pain?" And does not this again agree with what Le Dran has said in another part of the Memoir ‡ just now re-

* Mem. de l'Acad. de Chir. Tome III.

† Treatise of Operations, Chap. 26.

‡ Sect. 2, p. 32.

ferred ferred to, and with what has been since observed by others *, and by Albucasis † long before?

*Stroud-Water*,

November 25, 1788.

* Hill's Cases in Surgery, p. 21. — Justamond. Account of the Methods pursued in the Treatment of cancerous Disorders, &c., p. 132.—Kirkland. Thoughts on Amputation, p. 52.—Fearon. Treatise on Cancers, Case 8.

† Freind's History of Physic, Vol. II. p. 150.

